# Semi‐Supervised Learning Allows for Improved Segmentation With Reduced Annotations of Brain Metastases Using Multicenter MRI Data

**DOI:** 10.1002/jmri.29686

**Published:** 2025-01-10

**Authors:** Jon André Ottesen, Elizabeth Tong, Kyrre Eeg Emblem, Anna Latysheva, Greg Zaharchuk, Atle Bjørnerud, Endre Grøvik

**Affiliations:** ^1^ Computational Radiology and Artificial Intelligence (CRAI) Research Group, Division of Radiology and Nuclear Medicine Oslo University Hospital Oslo Norway; ^2^ Department of Physics, Faculty of Mathematics and Natural Sciences University of Oslo Oslo Norway; ^3^ Department of Radiology Stanford University Stanford California USA; ^4^ Department of Physics and Computational Radiology, Division of Radiology and Nuclear Medicine Oslo University Hospital Oslo Norway; ^5^ Institute of Clinical Medicine, Faculty of Medicine University of Oslo Oslo Norway; ^6^ Division of Radiology and Nuclear Medicine Oslo University Hospital Oslo Norway; ^7^ Department of Radiology Ålesund Hospital, Møre og Romsdal Hospital Trust Ålesund Norway; ^8^ Department of Physics Norwegian University of Science and Technology Trondheim Norway

**Keywords:** deep learning, brain metastases, segmentation, semi‐supervised

## Abstract

**Background:**

Deep learning‐based segmentation of brain metastases relies on large amounts of fully annotated data by domain experts. Semi‐supervised learning offers potential efficient methods to improve model performance without excessive annotation burden.

**Purpose:**

This work tests the viability of semi‐supervision for brain metastases segmentation.

**Study Type:**

Retrospective.

**Subjects:**

There were 156, 65, 324, and 200 labeled scans from four institutions and 519 unlabeled scans from a single institution. All subjects included in the study had diagnosed with brain metastases.

**Field Strength/Sequences:**

1.5 T and 3 T, 2D and 3D T1‐weighted pre‐ and post‐contrast, and fluid‐attenuated inversion recovery (FLAIR).

**Assessment:**

Three semi‐supervision methods (mean teacher, cross‐pseudo supervision, and interpolation consistency training) were adapted with the U‐Net architecture. The three semi‐supervised methods were compared to their respective supervised baseline on the full and half‐sized training.

**Statistical Tests:**

Evaluation was performed on a multinational test set from four different institutions using 5‐fold cross‐validation. Method performance was evaluated by the following: the number of false‐positive predictions, the number of true positive predictions, the 95th Hausdorff distance, and the Dice similarity coefficient (DSC). Significance was tested using a paired samples *t* test for a single fold, and across all folds within a given cohort.

**Results:**

Semi‐supervision outperformed the supervised baseline for all sites with the best‐performing semi‐supervised method achieved an on average DSC improvement of 6.3% ± 1.6%, 8.2% ± 3.8%, 8.6% ± 2.6%, and 15.4% ± 1.4%, when trained on half the dataset and 3.6% ± 0.7%, 2.0% ± 1.5%, 1.8% ± 5.7%, and 4.7% ± 1.7%, compared to the supervised baseline on four test cohorts. In addition, in three of four datasets, the semi‐supervised training produced equal or better results than the supervised models trained on twice the labeled data.

**Data Conclusion:**

Semi‐supervised learning allows for improved segmentation performance over the supervised baseline, and the improvement was particularly notable for independent external test sets when trained on small amounts of labeled data.

**Plain Language Summary:**

Artificial intelligence requires extensive datasets with large amounts of annotated data from medical experts which can be difficult to acquire due to the large workload. To compensate for this, it is possible to utilize large amounts of un‐annotated clinical data in addition to annotated data. However, this method has not been widely tested for the most common intracranial brain tumor, brain metastases. This study shows that this approach allows for data efficient deep learning models across multiple institutions with different clinical protocols and scanners.

**Level of Evidence:**

3

**Technical Efficacy:**

Stage 2

Brain metastases are the most prevalent intracranial tumor, and most commonly originate from lung cancer (20%–56%), breast cancer (5%–20%), or malignant melanomas (7%–16%).^1^, ^2^ The reported incidence of brain metastases is also increasing.^3^ High resolution 3D contrast‐enhanced T_1_‐weighted MRI is the recommended modality for assessment of tumor status and/or treatment response.^4^ The imaging assessment of brain metastases is a tedious and time‐consuming task, including lesion detection, delineation of the tumor area for radiotherapy, and subsequent measurement for tumor progression.^5^, ^6^ The time‐consuming nature of tumor delineation is evident in the recommendations of the Response Assessment in Neuro‐Oncology (RANO) group, where they advocate for unidimensional measurements rather than volumetric measurements due to the additional burden during radiological assessment.^6^ In recent years, substantial efforts have been made to reduce the burden placed on radiologists through automatic segmentation of brain metastases using deep learning models.^7^, ^8^, ^9^, ^10^, ^11^ Many of these 3D models are trained in a supervised manner on pairs of volumetric MR images with the corresponding human‐created tumor delineation masks.^8^, ^12^, ^13^, ^14^

Semi‐supervised learning is a subset of methods that combine annotated and unlabeled data to achieve satisfactory performance via an annotation‐efficient manner.^15^ Various strategies have been employed to leverage semi‐supervised learning including (I) pseudo‐labeling which refines the model using predictions on an unlabeled dataset, and (II) mean teacher which enforces consistency between a student and teacher network.^16^, ^17^ Despite the promising potential of semi‐supervised learning and the recent availability of public brain metastases datasets, its application for brain metastases segmentation remains largely unexplored.^18^, ^19^ A limited number of studies report using semi‐supervised learning and self‐supervised training for this task.^18^, ^20^ Nonetheless, semi‐supervised learning has shown great capabilities for neuro MRI and medical imaging.^21^, ^22^, ^23^

This study aimed to investigate whether semi‐supervised learning can improve segmentation performance and reduce the need for expert annotations of brain metastases. The three methods adopted are so‐called “consistency regularization methods,” where the overarching aim is to ensure consistency across models and/or perturbed variations of the same image. Mean teacher (MT) aims to achieve consistency by minimizing the mean squared error between the outputs from a student model and the exponentially moving average of the student models' weights, i.e., the teacher model.^17^ The second method, cross‐pseudo supervision (CPS), two models are trained simultaneously in a supervised manner, and the models use the other models' output from the labeled and unlabeled data as an additional ground truth besides the annotations.^24^ Interpolation consistency training (ICT) enforces consistency between the linear combination of two predictions from two images and the prediction of a linear combination of the same two images.^25^ The three methods were compared to a supervised baseline using 5‐fold cross‐validation, and evaluated on a multicenter dataset from four independent institutions.^7^, ^26^, ^27^, ^28^

## Materials and Methods

### Datasets

The study was approved by the Regional Medical Ethics Committee for Oslo University Hospital (OUH) and the Institutional Review Board at Stanford University.

This study included data from four separate centers all with confirmed brain metastases: Stanford University, OUH, University of California, San Francisco (UCSF), and Yale New Haven Hospital (YNHH).^27^, ^28^ The Stanford dataset consisted of two separate datasets: a labeled set with 156 examinations, and an unlabeled set with 519 examinations. All examinations were examined with the following image protocol: inversion recovery fast spoiled gradient echo (BRAVO), pre‐ and post‐contrast T1‐weighted sequence, and a 3D fluid attenuated inversion recovery sequence (FLAIR). The annotated Stanford data were split randomly into a training and test dataset containing 105 and 51 cases, respectively. All annotated data were sourced from different, unique pre‐treatment patients (105 female, 51 male), while some of the unlabeled dataset included pre‐ and/or post‐treatment follow‐up scans from 261 patients (168 female, 94 male). The mean age for the annotated Stanford cohort was 64 years (32–92) and 61 years (32–92) for the unlabeled cohort.

The OUH cohort comprised 65 patients (35 female/30 male) eligible for stereotactic radiotherapy with pre‐ and post‐contrast T1‐weighted fast spin echo (SPACE), and a 3D FLAIR. The mean age for the OUH cohort was 65 years (32–86).

The UCSF and YNHH datasets include 324 and 200 annotated examinations with T1 pre‐ and post‐contrast and FLAIR. Further details may be found in the original works.^27^, ^28^ The brain tumor segmentation (BraTS‐METS) masks with necrosis and enhancement were used as the ground truth for UCSF dataset.^19^

Annotations for the Stanford dataset was provided by two neuroradiologists with 2 (E.T.) and 8 (M.I.) years of experience (enhancing and necrotic tissue).^7^ The OUH annotations were made by two neuroradiologists with 5 (A.L.) and 14 (C.S.) years of experience (enhancing and necrotic tissue).^26^ The UCSF dataset was annotated by two neuroradiology fellows with 4 (L.P.S.) and 5 (J.E.V.) years of experience (enhancing and necrotic tissue).^28^ The YNHH dataset was annotated by a medical student (L.J.) and verified by two board‐certified neuroradiologists (M.S.A. and F.M.) with over 7 years of experience.^27^ The Stanford, UCSF, and YNHH datasets are publicly available and are available from their respective works.^7^, ^27^, ^28^ The OUH dataset and the unlabeled data from Stanford are available from the corresponding author upon reasonable request. The sequences and the key scan parameters are detailed in Table 1 for the unlabeled dataset from Stanford University. The imaging parameters for the other cohorts are detailed in their respective works.

**TABLE 1:** Overview of the MRI Sequences and the Most Common Imaging Parameters for the Unlabeled Dataset From Stanford University

Parameter	3D T1 BRAVO	Pre‐/Post‐3D T1 CUBE	3D CUBE FLAIR
TR (msec)	8.24	600	6000
TE (msec)	3.24	11	119
Flip angle	13	90	90
Inversion time (msec)	400	‐	1700
Pixel spacing	0.4688 × 0.4688	0.5 × 0.5	0.4688 × 0.4688
Slice thickness (mm)	1.0	1.0	1.2
Acquisition plane	Axial	Sagittal	Sagittal

### Study Overview

**FIGURE 1:**
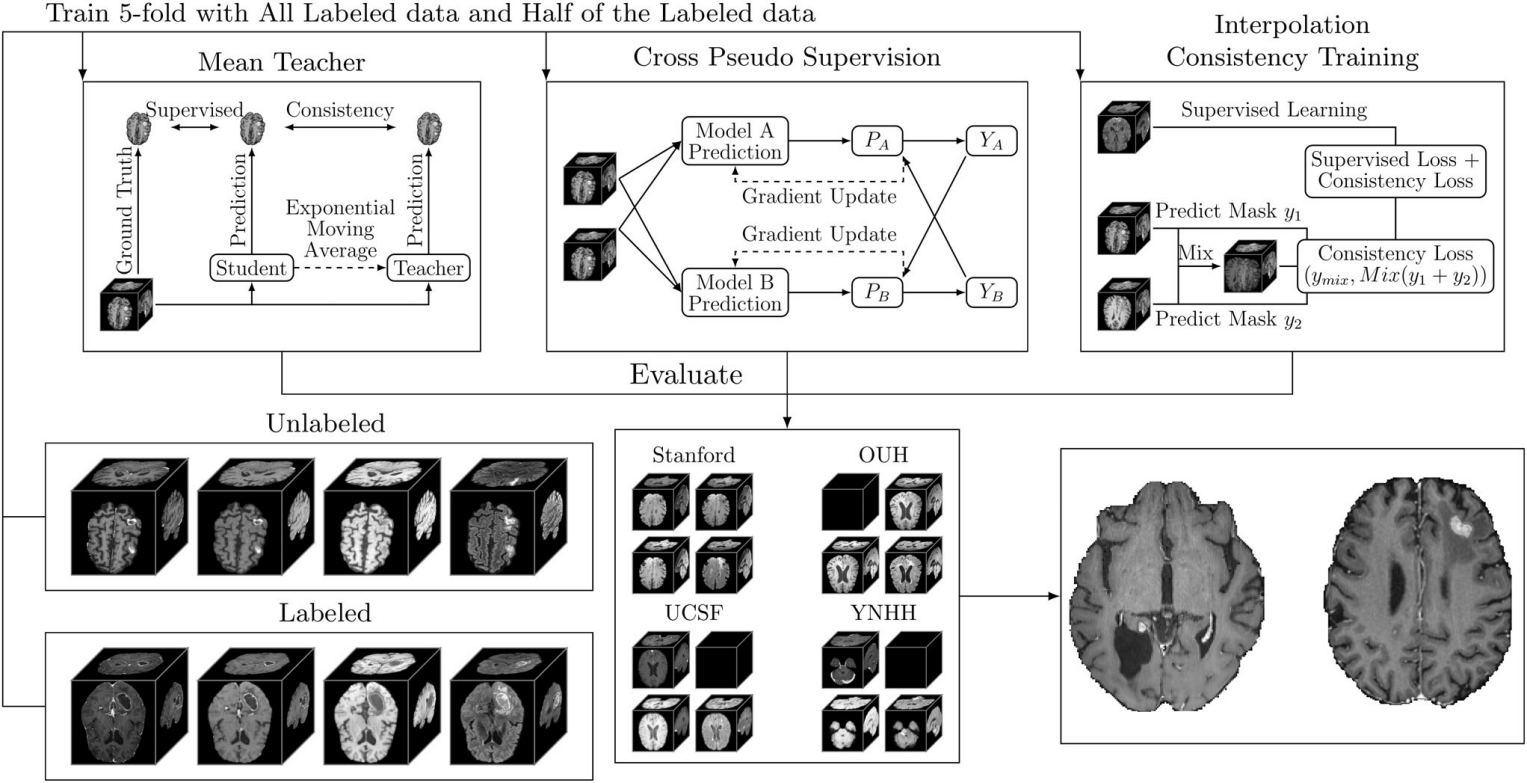
An overview of the study. A dataset that consists of labeled and unlabeled data were used to train segmentation models using three semi‐supervised methods, mean teacher, cross‐pseudo supervision, and interpolation consistency training with 5‐fold cross‐validation besides supervised training. All models from the 5‐fold cross‐validation were evaluated on an in‐house test set and three additional test sets from different institutions. The supervised training was not included to improve readability. Black boxes indicate that a sequence was not available during inference, i.e., the OUH cohort did not include the BRAVO sequence while the UCSF and YNHH did not include the CUBE sequence.

Three semi‐supervised methods: mean teacher (MT), cross‐pseudo supervision (CPS), and interpolation consistency training (ICT) were evaluated against a fully supervised baseline.^17^, ^24^, ^25^ The methods were trained with two different amounts of labeled data; the full dataset with 105 labeled examinations and half the data with 52 labeled examinations randomly selected from the 105 examinations, in both cases, 519 unlabeled examinations were used for semi‐supervision. Twenty percent of the labeled cases were withheld for model validation, i.e., 21 or 11 examinations were used for validation. All methods were trained with 5‐fold cross‐validation, as such the 21/11 validation cases differed for each fold. All semi‐supervised and supervised folds were evaluated on 51 hold‐out cases from Stanford, and three independent patient cohorts with 65 examinations (65 patients) from OUH, 324 examinations (154 patients) from the publicly available UCSF dataset,^28^ and 200 (200 patients) examinations from the publicly available YNHH dataset.^27^ An overview of the study design and the semi‐supervised methods is presented in Fig. 1. In addition, to evaluate generalizability when trained on different data. The best‐performing method trained on the Stanford dataset was re‐trained with 5‐fold cross‐validation on the UCSF dataset with 40/10 as the train/validation set, 75 patients withheld for evaluation, and the remaining 199 cases were used as an unlabeled set.

### Preprocessing

All MRI scans in an examination were coregistered to the T1 post‐contrast BRAVO sequence for the Stanford data, and to the post‐contrast T1‐weighted sequence for the OUH data, respectively. Coregistration was performed using the nordicICE software package (NordicImagingLab, Bergen, Norway) and advanced normalization tools (ANTs).^29^ Co‐registration was not performed on the UCSF and YNHH cohorts since the dataset available was already co‐registered. Brain extraction was performed using HD‐BET.^30^ The brain extracted image‐series were rescaled to an isotropic voxel size of 1×1×1 mm^3^ using trilinear interpolation, and the annotations were rescaled by nearest neighbor interpolation.

### Training

All methods (supervised and semi‐supervised) used the same 3D U‐Net architecture, configured similarly to nnUNet^30^ in the MONAI framework with four input channels (BRAVO, T1 CUBE post‐contrast, T1 CUBE pre‐contrast, and FLAIR).^31^ All models were trained for 1000 epochs, with each epoch consisting of 250 iterations (i.e., 250 batches) with a compound loss function combining Dice loss and binary cross‐entropy weighting metastases voxels 10‐fold due to data imbalance. The AdamW optimizer^32^ was used with learning rates of 1e−4 and 2e−4 for the supervised and semi‐supervised models, with an initial linear warm‐up period the first 50 epochs followed by cosine annealing learning rate scheduling. The semi‐supervised methods had a batch size of four, comprising two labeled and two unlabeled samples while the supervised baseline had a batch size of two. The epoch with the lowest validation loss was selected for inference for each fold on all methods‐ including the supervised baseline and all semi‐supervised models.

Data augmentation was used on the labeled and unlabeled data across all models. This included random flipping, rescaling by a random factor within the range of [0.85, 1.25], and rotation to mimic head displacement. Input level dropout was used with a 25% chance to drop any of the four input sequences to allow for models to accept a varied number of input sequences.^26^

Model evaluation metrics included 1) the Dice similarity coefficient (DSC), 2) the 95 percentile Hausdorff distance (HD95), 3) the number of correctly predicted metastases, and 4) the number of false positive metastases. An overlap of 10% between the predicted metastases and the ground truth metastases was considered a correctly predicted metastasis.^7^ The predictions were thresholded with separate values that maximized the DSC on the validation dataset for each fold.

In the CPS‐generated confidence maps, a threshold factor of 0.5 was applied to create binary masks during model training on the unlabeled dataset. Thresholds of 0.1, 0.9, and no threshold were also tested, but a threshold of 0.5 showed the best performance during initial testing. The training code and all hyperparameters are detailed in the GitHub repository.^1^

A paired samples two‐sided *t* test was used to compare the supervised and semi‐supervised methods within each fold for each dataset, and across the means for the folds for a given method. A Bonferroni correction of 4 was used when comparing a single method across multiple datasets, and a Bonferroni correction of 5 was used when each fold was considered separately.

## Results

**FIGURE 2:**
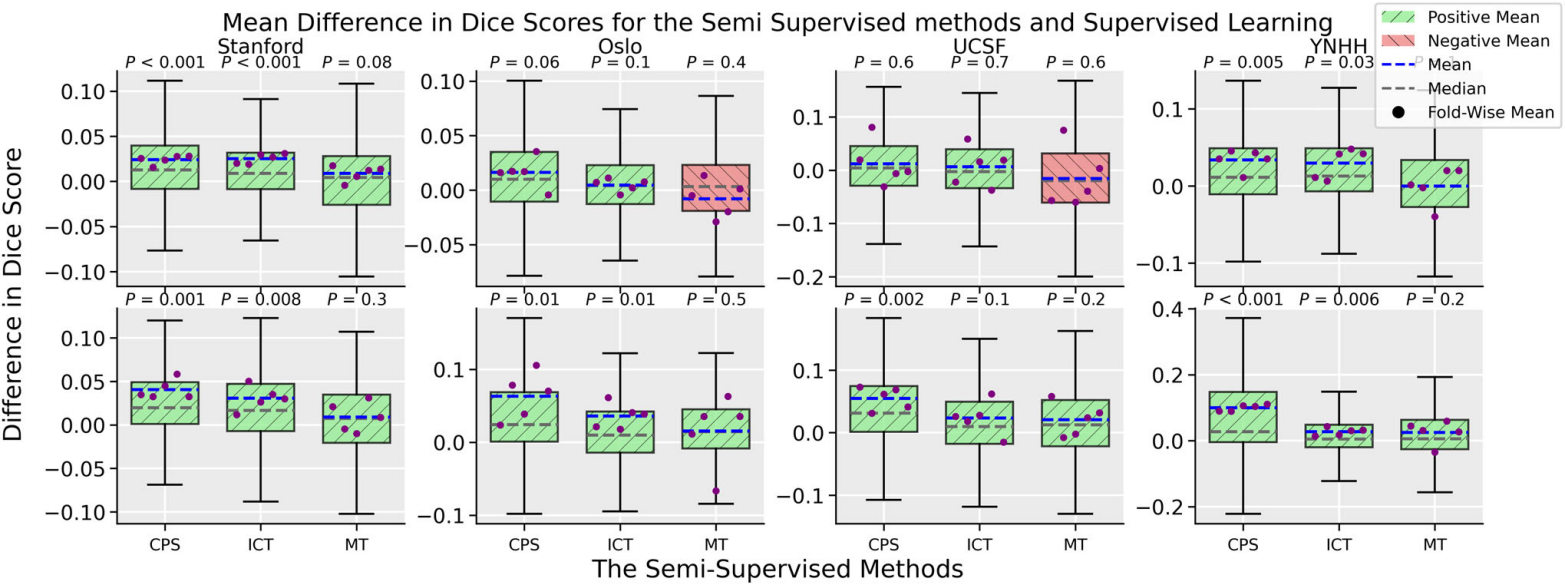
The mean difference in Dice similarity averaged across the five folds for their respective amount of labeled data between the supervised training regime and the three semi‐supervised methods, cross‐pseudo supervision (CPS), interpolation consistency training (ICT), and mean teacher (MT) for Stanford, Oslo University Hospital (OUH), University California San Fransisco (UCSF), and Yale New Haven Hospital (YNHH) datasets with the full training dataset (top row) and half the training data (bottom row). Outliers were excluded to help readability; an improved DSC between the semi‐supervised method is highlighted with green, and a decrease in the DSC is highlighted with red.

Table 2 presents, and Fig. 2 depicts the difference in DSC and the *P*‐values for the supervised, MT, CPS, and ICT methods on the Stanford, OUH, UCSF, and YNHH test datasets, respectively, with the full and half sized training dataset averaged across the five folds. Across all folds and amount of training data, semi‐supervised training showed improved performance for 36/40, 36/40, and 23/40 cases for CPS, ICT, and MT, respectively. CPS and ICT showed a consistent improved DSC compared to the supervised baseline across all datasets and training data amount where 36/40 cases showed improved performance. Across all test sets, the improvement seen was in the range of 6.3%–15.4% for CPS (*P* < 0.0125 on all test cohorts) and 3.8%–4.8% for ICT (*P* < 0.0125 on 3/4 test cohorts) when trained on half the data, and 1.8%–9.2% for CPS (*P* < 0.0125 on 2/4 test cohorts) and 0.6%–6.8% for ICT (*P* < 0.0125 on 1/4 test cohorts) when trained on the full dataset. Notably, the semi‐supervised models from CPS and ICT when trained on half the training data had a DSC that was equal or close to the fully supervised baseline trained on twice the labeled sample size. There was a noticeably larger relative and absolute improvement between the semi‐supervised and supervised models for the independent test datasets when trained with less data (8.2% ± 3.8%, 8.6% ± 2.6%, and 15.4% ± 1.4% compared to 6.3% ± 1.6% for the CPS method); contrary, when trained on more labeled data, the semi‐supervised models exhibited less improvement than on the in‐house dataset (1.8% ± 5.7%, 2.0% ± 1.5%, and 4.7% ± 1.7% compared to 3.6% ± 0.7% for the CPS method). Of all methods tested, MT showed the smallest improvement, and in two cases showed a decrease in the DSC compared to the supervised baseline (1.5% ± 2.4% compared to 6.3% ± 1.6% and 4.8% ± 1.9% on the Stanford dataset when trained on half the labeled dataset) with *P* = 0.3. Table 3 details the 95HD for the supervised baseline and semi‐supervised methods across all training dataset sizes and test datasets. The 95HD shows a similar sentiment as seen in the DSCs with the 95HD showing a decrease between −1.5% and −33.5% when the models were trained on half the labeled dataset across all tested semi‐supervised methods (2/4 test sets had *P* < 0.0125 for the CPS method, 2/4 test sets had *P* < 0.0125 for the ICT method, and 1/4 test sets had *P* < 0.0125 for the MT method). In addition, the improvement between the supervised baseline and the semi‐supervised methods decreased when the amount of labeled data increased: −0.6% to −16.5% (with a single case having a decreased performance of 7.4%).

**TABLE 2:** The Mean and Standard Deviation of the Dice Similarity Coefficient for the Supervised, Mean Teacher (MT), Cross‐Pseudo Supervision (CPS), and Interpolation Consistency (ICT) Based Methods Averaged Across the Five Folds on the Test Datasets With the Full and Half Training Dataset

	Supervised	MT	%	CPS	%	ICT	%
Full dataset							
Stanford	0.66 ± 0.01	**0.67 ± 0.01**	**1.4 ± 1.2**	**0.68 ± 0.01**	**3.6 ± 0.7**	**0.68 ± 0.01**	**3.8 ± 0.7**
Oslo	0.80 ± 0.01	0.79 ± 0.01	−1.0 ± 1.9	**0.82 ± 0.01**	**2.0 ± 1.5**	**0.81 ± 0.01**	**0.6 ± 0.7**
UCSF	0.67 ± 0.03	0.65 ± 0.03	−2.3 ± 7.7	**0.68 ± 0.02**	**1.8 ± 5.7**	**0.67 ± 0.02**	**1.1 ± 5.1**
Yale	0.70 ± 0.03	**0.70 ± 0.02**	−0.2 ± 3.2	**0.73 ± 0.02**	**4.7 ± 1.7**	**0.73 ± 0.01**	**4.2 ± 2.5**
Half dataset							
Stanford	0.62 ± 0.01	**0.63 ± 0.02**	**1.5 ± 2.4**	**0.66 ± 0.01**	**6.3 ± 1.6**	**0.65 ± 0.01**	**4.8 ± 1.9**
Oslo	0.75 ± 0.02	**0.76 ± 0.03**	**2.0 ± 6.0**	**0.81 ± 0.01**	**8.2 ± 3.8**	**0.78 ± 0.01**	**4.7 ± 2.1**
UCSF	0.61 ± 0.01	**0.63 ± 0.02**	**3.3 ± 3.8**	**0.67 ± 0.01**	**8.6 ± 2.6**	**0.64 ± 0.02**	**3.8 ± 3.9**
Yale	0.60 ± 0.01	**0.63 ± 0.03**	**4.0 ± 5.3**	**0.70 ± 0.01**	**15.4 ± 1.4**	**0.63 ± 0.01**	**4.5 ± 1.7**

The percentage listed is the relative difference between the supervised and the semi‐supervised methods. Cases with a higher Dice similarity coefficient compared to the supervised baseline are highlighted in bold.

**TABLE 3:** The 95HD Distance for the Supervised, Mean Teacher (MT), Cross‐Pseudo Supervision (CPS), and Interpolation Consistency (ICT) Based Methods Averaged Across the Five Folds on the Test Datasets With the Full and Half Training Dataset

	Supervised	MT	%	CPS	%	ICT	%
Full dataset							
Stanford	21.6 ± 2.3	**21.3** ± 1.7	**−0.9**	**20.7** ± 1.9	**−4.0**	**20.7** ± 2.2	**−4.0**
Oslo	11.1 ± 1.7	11.9 ± 2.0	7.4	**10.8** ± 1.5	**−1.6**	**11.0** ± 0.5	**−0.6**
UCSF	26.5 ± 2.4	**24.8** ± 1.2	**−6.5**	**26.0** ± 5.6	**−3.7**	**23.2** ± 1.6	**−13.3**
Yale	27.7 ± 5.4	**24.5** ± 2.3	**−10.3**	**26.6** ± 5.4	**−3.9**	**23.2** ± 3.1	**−16.5**
Half dataset							
Stanford	21.8 ± 0.4	**21.7** ± 2.9	**−1.5**	**19.2** ± 1.4	**−13.0**	**17.6** ± 2.0	**−22.2**
Oslo	14.3 ± 1.7	**12.8** ± 1.2	**−10.8**	**10.1** ± 0.6	**−33.5**	**11.9** ± 1.9	**−18.9**
UCSF	31.4 ± 4.3	**25.7** ± 2.7	**−19.6**	**25.5** ± 1.7	**−20.0**	**26.0** ± 1.6	**−19.1**
Yale	32.3 ± 1.7	**26.7** ± 1.7	**−19.0**	**25.5** ± 2.2	**−23.9**	**28.0** ± 2.5	**−14.6**

The percentage listed is the relative difference between the supervised and the semi‐supervised methods. Cases with a lower HD95 compared to the supervised baseline are highlighted in bold.

**FIGURE 3:**
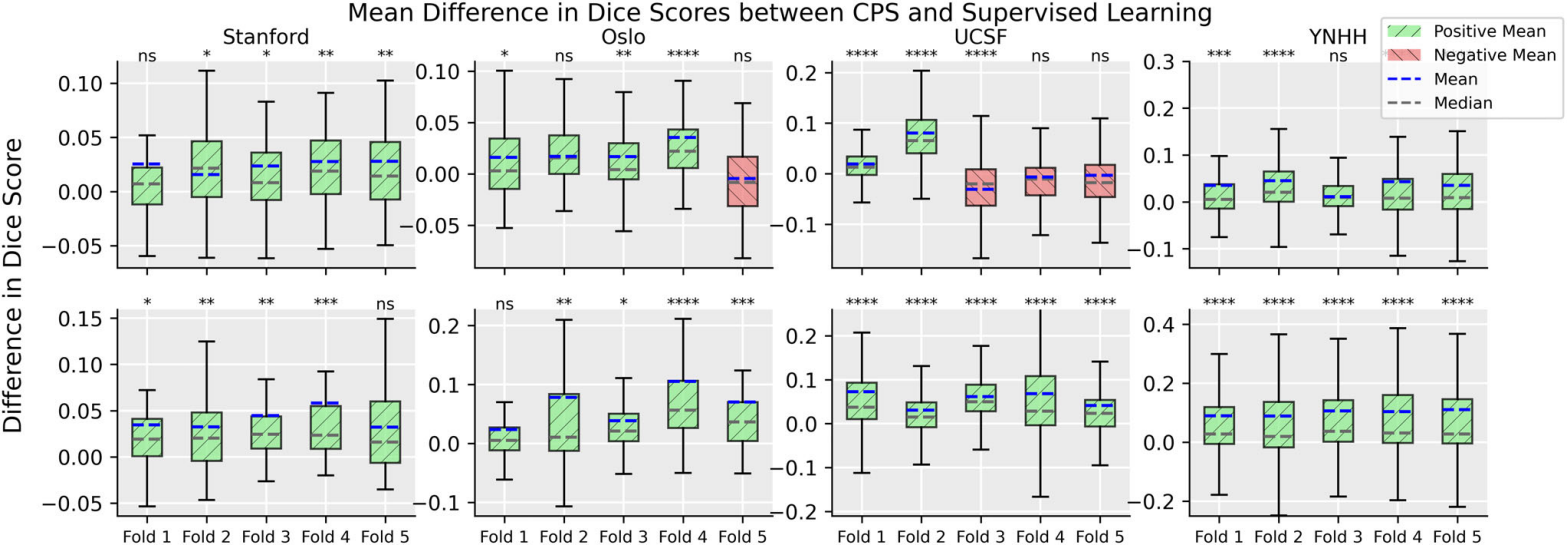
The mean difference in Dice similarity coefficient (DSC) for the five folds for their respective amount of labeled data between the supervised training regime and cross‐pseudo supervision for Stanford, Oslo University Hospital (OUH), University California San Fransisco (UCSF), and Yale New Haven Hospital (YNHH) datasets with the full training dataset (top row) and half the training data (bottom row). Outliers were excluded to help readability; an improved DSC between the semi‐supervised method is highlighted in green, and a decrease in the DSC is highlighted with red. To improve readability the exact *P*‐values are not listed, instead it follows the following convention; ns = *P* ≥ 0.05; * = 0.01 < *P* < 0.05; ** = 0.005 < *P* ≤ 0.01; *** = 0.001< *P* ≤ 0.005; **** = *P* ≤ 0.001. Note, a Bonferroni correction of 5 is used to determine significance.

**FIGURE 4:**
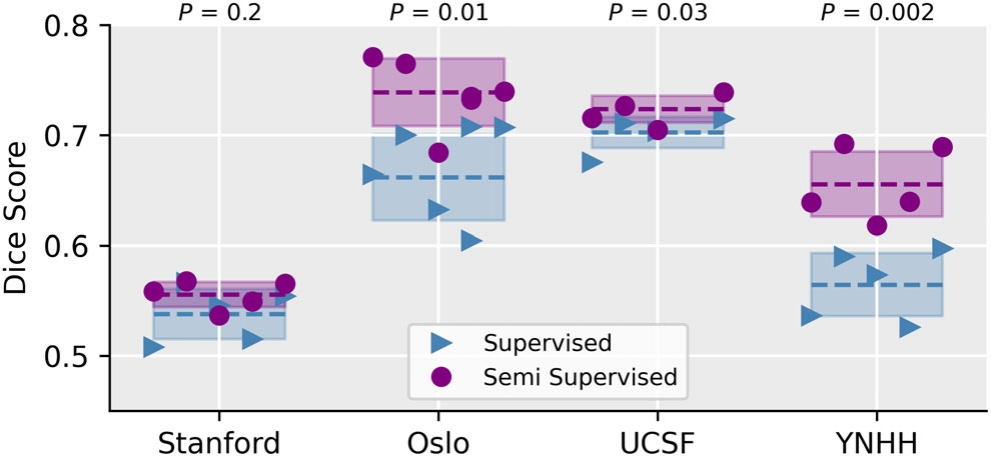
The mean Dice similarity coefficient (DSC) for each of the five folds for the supervised baseline and the cross‐pseudo supervision (CPS) semi‐supervised method when trained on the University California San Fransisco (UCSF) dataset. Evaluation was performed on three withheld datasets from Stanford, Oslo University Hospital (OUH), Yale New Haven Hospital (YNHH), and a withheld test set from the UCSF cohort.

Figure 3 shows the difference in DSC and indicates the *P*‐value between the supervised baseline and the CPS method across all datasets and folds when training with full and half training dataset. There is a consistent improvement for all test datasets and training set size with 5/10, 5/10, and 9/10 evaluations for Stanford, OUH, and YNHH, respectively, had an improvement with *P* < 0.01 over all folds except the UCSF dataset when the models were trained on the full training data. A similar trend was observed for ICT. Semi‐supervision gives consistent and noticeable improvement on independent datasets when trained on few samples, but the effect lessens with more training data. MT did not exhibit any notable improvement on the independent datasets when trained on the full training dataset. All DSCs, the 95HD, *P*‐values per fold and across folds, and the effect size is given in the Supplemental Materials. Figure 4 shows the DSC for CPS and the supervised baseline when trained on 50 samples from the UCSF data and evaluated on all test cohorts. Similar to the results from the models trained on the Stanford dataset, there is a consistent improvement over the supervised baseline of 3.3% ± 4.1%, 11.1% ± 5.4%, 3.0% ± 1.8%, and 14.9% ± 4.1% for Stanford, OUH, UCSF, and YNHH, respectively (with YNHH have *P* < 0.0125).

**FIGURE 5:**
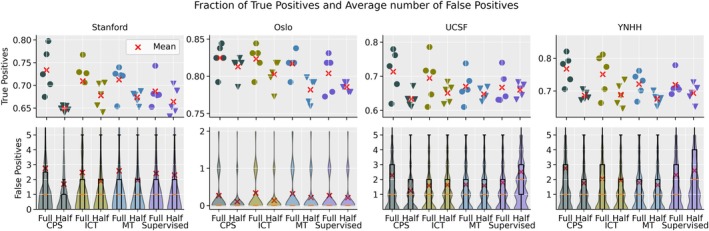
The faction of true positive metastases predictions and the average number of false positives for the Stanford, Oslo University Hospital (OUH), University California San Fransisco (UCSF), and Yale New Haven Hospital (YNHH) test datasets for all 5‐folds trained supervised and semi‐supervised with cross‐pseudo supervision (CPS), mean teacher (MT), and interpolation consistency training (ICT).

The average number of correctly predicted metastases and the average number of false positives for the five folds is detailed in Table 4 for the supervised baseline and the semi‐supervised methods across the four test datasets. There is no notable increase in the number of correctly predicted brain metastases except for YNHH dataset. Figure 5 shows the fraction of true positives for each fold separately and violin plots of the number of false positives for the Stanford, OUH, UCSF, and YNHH cohorts for all methods. When trained on the full labeled dataset, the true positives of all three semi‐supervised models were slightly better than supervised model in all four cohorts (Stanford, OUH, UCSF, and YNHH). Concomitant false positives were only marginally increased in the Stanford cohort (2.5–2.8 compared with 2.4 in supervised model), in the UCSF CPS model (2.3 compared with 1.8 in supervised model), and the YNHH (1.8–2‐8 compared with 2.3 in the supervised model) while they remained unchanged or close to unchanged in the OUH cohort. When trained with half training data, the improvement in true positives was observed in fewer semi‐supervised models, namely, ICT and MT models with the Stanford cohort, ICT and CPS models with the OUH cohort. However, the concomitant false positives were reduced in all three semi‐supervised models in all four cohorts except for MT in the OUH cohort.

**TABLE 4:** The Number of Correctly Predicted and the Number of False Positive Brain Metastases for the Supervised, Mean Teacher (MT), Cross‐Pseudo Supervision (CPS), and Interpolation Consistency (ICT) Based Methods Averaged Across the Five Folds on the Test Datasets With the Full and Half Training Dataset

	Supervised	MT	CPS	ICT
True positives				
Full dataset				
Stanford (864)	69% ± 3% (594)	**71% ± 3% (616)**	**73% ± 4% (634)**	**71% ± 5% (613)**
Oslo (154)	80% ± 2% (124)	**82% ± 1% (126)**	**82% ± 2% (127)**	**82% ± 2% (127)**
UCSF (3341)	67% ± 4% (2229)	**67% ± 4% (2238)**	**71% ± 6% (2384)**	**69% ± 6% (2321)**
Yale (999)	72% ± 3% (718)	**72% ± 3% (721)**	**77% ± 4% (767)**	**75% ± 6% (750)**
Half dataset				
Stanford (864)	66% ± 3% (574)	**67% ± 1% (**582)	65% ± 1% (561)	**68% ± 3% (585)**
Oslo (154)	79% ± 1% (121)	78% ± 2% (120)	**81% ± 1% (125)**	**80% ± 2% (124)**
UCSF (3341)	66% ± 2% (2206)	65% ± 2% (2164)	63% ± 2% (2116)	65% ± 3% (2175)
Yale (999)	69% ± 3% (693)	68% ± 2% (675)	69% ± 1% (685)	69% ± 3% (688)
False positives				
Full dataset				
Stanford	2.4 ± 0.8	2.6 ± 0.6	2.8 ± 1.0	2.5 ± 0.9
Oslo	0.3 ± 0.1	0.3 ± 0.1	**0.3 ± 0.2**	0.3 ± 0.2
UCSF	1.8 ± 0.8	**1.7 ± 0.6**	2.3 ± 1.4	**1.6 ± 0.8**
Yale	2.3 ± 1.2	**1.8 ± 0.7**	2.8 ± 1.6	**2.1 ± 1.2**
Half dataset				
Stanford	2.3 ± 0.4	**1.9 ± 0.6**	**1.7 ± 0.3**	**1.9 ± 0.3**
Oslo	0.2 ± 0.1	0.2 ± 0.1	**0.1 ± 0.04**	**0.1 ± 0.06**
UCSF	2.5 ± 0.6	**1.6 ± 0.5**	**1.2 ± 0.5**	**1.6 ± 0.4**
Yale	2.6 ± 0.5	**1.6 ± 0.3**	**1.8 ± 0.5**	**1.9 ± 0.3**

Cases with a higher number of true positives or lower number of false positives compared to the supervised baseline are highlighted in bold.

**FIGURE 6:**
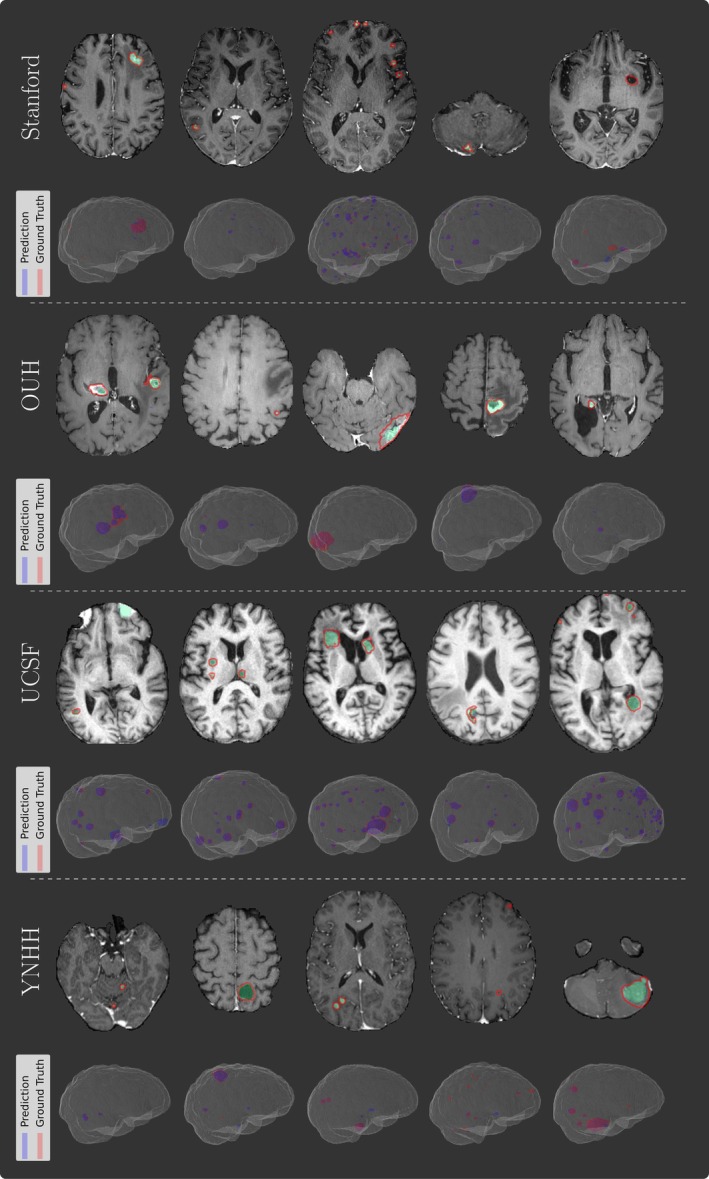
Examples of model predictions using cross‐pseudo supervision with the full training dataset from fold three on the Stanford, Oslo University Hospital (OUH), University California San Fransisco (UCSF), and Yale New Haven Hospital (YNHH) test datasets. The top row for each dataset is an axial slice where the ground truth is shown by the red outline, and the prediction is shown by the heatmap. The bottom row is the corresponding volumetric prediction from the same examination as the slice.

Figure 6 showcases representative examples from the CPS‐based method, which achieved the highest DSC on the Stanford cohort. Of note, the smaller metastases proved problematic to accurately segment across all models. There are a couple of notable traits exhibited by most models: 1) there were more hyperintense extra‐cranial structures remaining after brain extraction in the UCSF dataset, as demonstrated in row five column one in Fig. 6; and 2) the mean number of false positives per patient is skewed by outlier patients with many false positives and the median value of 0 or 1 is more representative of the overall performance.

## Discussion

This study evaluated the feasibility of semi‐supervised learning for brain metastases segmentation by testing three commonly used semi‐supervised methods: mean teacher, cross‐pseudo supervision, and interpolation consistency training. The results suggests that semi‐supervised learning outperforms traditional supervised learning for brain metastases segmentation, as evidenced by a mean increase in the DSC and decrease in the 95HD when trained on different amounts of training data. When applying the models to independent dataset from three other institutions, which varied in terms of both MR examination design and national treatment guidelines, semi‐supervised training showed greater improvements when trained on less annotated data, but smaller improvements when trained more annotated data for two of the cohorts and greater improvement on the YNHH cohort. This demonstrates that the gain from semi‐supervised training generalizes to data from other institutions, though the improvement is less stable when compared to the in‐house test dataset. Consistent with previous works,^20^ there was an improvement when semi‐supervised learning was used. In addition, there was a notable decrease in the number of false positives when the models were trained on half the training data. This was not seen when trained on a larger number of labeled samples. This may be attributed to the already competitive performance of the supervised baseline, particularly with regard to the number of false positives compared to previous works.^7^, ^8^, ^14^, ^33^ Still, we note that higher detection rates have been reported elsewhere in literature.^34^, ^35^

The results suggest that semi‐supervised learning improves the performance of the models relative to the supervised baseline. A small positive trend is observed for the number of correctly predicted metastases for the semi‐supervised models compared to the supervised baseline. Overall, the semi‐supervised models attained higher true positives than supervised model, with only a marginal increase in false positives observed. It is important to note that the improvement in true positives is close to decoupled from the number of false positives for the supervised method. This shows that the improvement in the sensitivity in detecting brain metastases was not accomplished at the expense of false detection.

In a clinical setting, semi‐supervised training offers multiple important improvements over the supervised baseline. The improvement in model performance can allow smaller institutions to train in‐house models without a degradation in the performance. In addition, since most clinical sites have multiple MRIs from different vendors and field strengths, the results from the external cohorts suggest that models trained with semi‐supervised learning may be more robust to scanner differences. However, this robustness needs further testing. Overall, the improved accuracy and robustness may allow for automated annotation and reduced burden for clinicians, even with models trained on few labeled samples due to resource constraints.

### Limitations

More extensive testing with more folds is needed to validate the improved performance attributed to semi‐supervised learning, as the number of models trained for this study was limited. This is especially true with regards to the *P*‐values listed for the DSC averaged across the fold. Second, the scarcity of labeled data in our dataset restricts our ability to fully examine how the sample size of labeled data might influence the observed improvements. Due to the sheer number of semi‐supervised methods, we opted for three commonly used methods, however, there may be other methods better suited for semi‐supervised segmentation of brain metastases. As such, the goal was to demonstrate that semi‐supervised learning offers a notable improvement in the segmentation of brain metastases, which is valuable for treatment planning and monitoring disease progression.

## Conclusion

Semi‐supervised learning yielded consistent improvement in the predictive performance of brain metastases segmentation, using test datasets featuring both similar and dissimilar imaging protocols to the training data. All the tested semi‐supervised methods showed an improvement over the supervised baseline in the DSC. Still, there was no notable reduction in the number of false positives or increase in the percentage of correctly predicted metastases.

## Conflict of Interest

E.G. and K.E.E. have intellectual property rights at NordicNeuroLab AS, Bergen, Norway. A.B. is shareholder in NordicNeuroLab AS, Bergen, Norway. G.Z. declares equity interest in Subtle Medical Inc., funding support from GE Healthcare, and consults for Biogen.

## Supporting information


**Data S1.** Supporting Information.

**Data S2.** Supporting Information.

**Data S3.** Supporting Information.

## Footnotes

^1^

https://github.com/JonOttesen/SemiMetSeg
